# Female perception of a partner’s mate value discrepancy and controlling behaviour in romantic relationships

**DOI:** 10.1007/s10211-016-0240-5

**Published:** 2016-10-13

**Authors:** Dariusz P. Danel, Agnieszka Siennicka, Kinga Glińska, Piotr Fedurek, Natalia Nowak-Szczepańska, Ewa A. Jankowska, Bogusław Pawłowski, Zdzisław Lewandowski

**Affiliations:** 1 Ludwik Hirszfeld Institute of Immunology and Experimental Therapy, Polish Academy of Sciences, Rudolfa Weigla 12, 53-114 Wroclaw, Poland; 2 Department of Physiology, Wroclaw Medical University, ul. T. Chalubinskiego 10, 50-368 Wroclaw, Poland; 3 Department of Life Sciences, University of Roehampton, Roehampton Lane, London, UK; 4 Department of Human Biology, University of Wroclaw, ul. Kuznicza 35, 50-138 Wroclaw, Poland; 5 Department of Human Biology, Chair of Physiotherapy Foundations, Faculty of Physiotherapy, University School of Physical Education in Wroclaw, al. I. J. Paderewskiego 35, 51-612 Wroclaw, Poland

**Keywords:** Mate retention behaviours, Controlling behaviour, Mate value, Mate value difference, Heterosexual romantic relationships

## Abstract

Mate value discrepancy (MVD) between heterosexual partners is an important factor influencing relationship satisfaction which, in turn, has an effect on the quality and the stability of the relationship. Therefore, partners’ involvement in mate retention behaviours, such as controlling behaviours, can be related to MVD and our study aims to determine whether such an association exists. In order to do so, we analysed female perception of MVD and their opinion regarding the intensity of controlling behaviours performed by themselves as well as their romantic partners. Female perception of the intensity of controlling behaviours performed by both partners was the highest in couples where a woman assesses her own mate value (MV) as higher than her partner’s MV and significantly different than in relationships where male MV exceeded those of the female. Our study also indicates that MVD should be taken into account when analysing sex differences in intensities of mate retention behaviours. Finally, we provide evidence supporting the significance of the relationship length for controlling behaviour intensity. Findings are discussed within an evolutionary perspective.

Mate guarding, a behavioural strategy aiming to maintain physical proximity to a reproductive partner, is widespread among animals and is exhibited mainly by males (reviews e.g. Birkhead and Møller 1992; Dixon 2012; Thornhill and Alcock 1983; Parker 1974; Grafen and Ridley 1983). It has been argued that male mate guarding is a behavioural adaptation against cuckoldry aiming to reduce the potential costs related to investments of time and resources in genetically nonrelated offspring. However, in species showing high levels of parental investment, such as humans, mate guarding is expected to evolve also in females as a response to partner’s infidelity and risk of losing partners’ resources and protection (Flinn 1988; Buss 2002; Buss and Shackelford 1997; Kardum 2006; Graham-Kevan and Archer 2009 and citations therein).

Human mate guarding or, in a broader sense, mate retention behaviours (see: Buss 1988) can be classified in terms of costs and benefits that the romantic partner bears and receives (Miner et al. 2009b). For instance, time monopolization and public derogations may be highly expensive for the partner in terms of his/her self-esteem and social support diminution. In contrast, giving expensive gifts or paying compliments may be beneficial for the partner as it may potentially increase partner’s relationship satisfaction and decrease the likelihood of partner’s infidelity or defection (Miner et al. 2009b).

Among the individual tactics that transfer the costs of mate retention to a partner are controlling behaviours. These behaviours may involve not only economic and emotional control, but also threats, intimidation and isolation of the partner (Graham-Kevan and Archer 2009, 2005). The previously mentioned forms of mate control, sometimes alongside physical violence, can be classified as direct forms of mate guarding (Graham-Kevan and Archer 2005, 2009). However, unlike physical aggression, which is often socially stigmatised, controlling tactics are relatively widespread in humans often applied in order to control partner’s life (Graham-Kevan and Archer 2009) and affect relationship quality.

Among the potential factors that may influence couple well-being are the mate values (MVs) of both partners (Nowak and Danel 2014). MV is often defined within a reproductive context (e.g. Symons 2005; Waynforth 2001) and refers to all phenotypic aspects that promote successful reproduction of an individual (Sugiyama 2005; Trivers 1972). In a broader perspective, the definition of MV covers not only physical but also psychological and social characteristics (Buss and Barnes 1986; Lippa 2007). In such a multidimensional sense, MV includes all attributes that may potentially affect individual mate choice and mate retention capabilities in a given time and context (Fisher et al. 2008).

In order to maximise reproductive success, both males and females aim to find and mate with a partner of the best available MV. Consequently, people are expected to form relatively homogenous relationships with respect to MV levels (Buss and Barnes 1986; Buss and Duntley 2006). However, the previously mentioned assumption does not exclude the possibility of forming romantic relationships between individuals exhibiting significant discrepancy in their MVs. For example, the MV discrepancy between partners may occur when a lower-MV individual deceives a potential partner regarding the actual level of resources accessible, life aspirations or number of previous partners (Haselton et al. 2005). Similarly, the discrepancy between partners’ MVs may also appear when the MV of one party significantly increases (e.g. by moving up the career ladder) or decreases (e.g. job loss, loss of fertility, chronic illness) (Buss and Duntley 2011). In such circumstances, the partner with higher MV may try to terminate the disadvantageous relationship, whereas the partner with lower MV may use mate retention tactics in order to prevent the potential breakdown of the relationship (Buss and Duntley 2011). Consequently, one could expect that controlling behaviour may be related not only to the individual’s absolute MV but also to the self-assessment of MV relative to the MV of the partner or, in other words, mate value discrepancy (MVD) between partners.

There is a substantial scarcity of studies attempting to address the effect of MVD on mate retention tactics. In one such attempt, Buss and Shackelford (1997) found that men married to women of relatively higher MV (younger and perceived by the partner as more attractive) more frequently employed several mate retention tactics such as emotional manipulation, commitment manipulation, possessive ornamentation and derogations. These finding are in line with the results of other study showing that women with higher multifaceted MV than their partners feel lower satisfaction from their relationships (Nowak and Danel 2014). A lower satisfaction of high-MV women may result from more intensive mate guarding and impression of being trapped in the current relationship (Nowak and Danel 2014). Indeed, a recent study confirmed that MVD may affect mate retention behaviours through the relationship satisfaction (Conroy-Beam et al. 2016). Taken together, the previously mentioned studies suggest that also the intensity of more specific negative forms of mate guarding such as controlling behaviours may be related to the difference between qualities of romantic partners.

In the present study, we refer to work by Graham-Kevan and Archer (2009) and aim to investigate whether MVD is related to the intensity of negative forms of mate retention tactics, such as controlling behaviours. We focus on women’s reports since, comparing to men, the relationship between mate value and particular forms of mate retention is insufficiently investigated in women (see Starratt and Shackelford 2012), which is quite surprising given that both sexes use negative tactics of mate guarding with similar intensity (Graham-Kevan and Archer 2009). In a similar vein, we analyse women’s assessments of their partners’ use of controlling behaviours and possible differences between intensities of mate guarding performed by both sexes.

As noted previously, being involved in a relationship with a partner who exhibits lower MV might be disadvantageous in terms of maximizing individual’s fitness. Thus, we predict that controlling behaviour would be applied less intensively by women involved in relationships with lower-MV partner compared to those who are in relationship with a partner exhibiting higher MV. Similarly, we predict that in couples where man has lower MV than his partner, women will perceive that their partners exhibit more control over them compared to women involved with man exhibiting higher MV values than their own. Furthermore, following results by Graham-Kevan and Archer (2009), we also predict that there will be no difference in the general intensity of controlling behaviours performed by men and women. However, after taking MVD into account, we hypothesise that in the MV discrepant relationships, it is the lower-MV partner who exerts more control over his/her partner.

In addition to the previously mentioned hypotheses, we also focus on a potential role of relationship length in partner control. This is because several authors suggested that relationship length may influence mate retention and guarding strategies (Buss and Shackelford 1997; Graham-Kevan and Archer 2009; Shackelford et al. 2006). In particular, Buss and Shackelford (1997) proposed that in long-lasting, hence putatively more secure, relationships, the intensity of mate retention (at least in men) would be lower. However, longer relationships are those with higher total both partners’ investments accumulated over time and relationship breakdown may shatter them. Moreover, a breakdown of long-lasting relationship may be especially costly for women, since loss of time devoted to unsuitable partner depletes time pool (limited by the menopause) available for successful mating and reproduction. Therefore, we hypothesise that using controlling behaviours in long-term couples would be a strategy used in order to prevent the loss of partner and secure relationship investments. Therefore, we predict that the length of the relationship should be positively related to the intensity of controlling behaviour.

## Method

### Participants

The study took place in Wroclaw (Poland). Study participants, mostly students, were selected from 200 women who simultaneously took part in a different project that focused on female conditional preferences for male morphology. The potential study participants were informed about the project through the project website and a local newspaper. Some students who took part in the project were awarded two credit points (ECTS), and the first 120 participants received a cosmetic set worth 15 euros (the cosmetic company sponsor had no interest, financial or otherwise, in our analysis and its interpretation).

For this study, we selected only women who were currently in a heterosexual relationship and completed all questions presented (*N* = 133). The mean age of this sample was 23.78 years (SD = 4.78) for females and 25.78 years (SD = 5.53) for their partners (partners’ mean age and SD calculated for *N* = 132 men, one value was missing). The mean length of the relationships of the women was 44.17 months (SD = 48.54), and the modal value was 18 months (range 2 to 264 months).

All study procedures were in accordance with the ethical standards of the institutional and/or national research committee and with the 1964 Helsinki Declaration and its later amendments or comparable ethical standards. Informed consent was obtained from all individual participants included in the study.

### Procedure

Each participant provided information regarding her age, the age of her partner and the duration of the relationship. The participants also completed a revised version of the Controlling Behaviour Scale (CBS-R) developed by Graham-Kevan and Archer (2005) and translated into Polish. The wording was kept as close as possible to the original, and only minor changes were introduced to adjust the questionnaire to Polish grammar and phraseology (translation available on request). In the questionnaire, subjects were asked to assess own and their own partner’s frequency of controlling behaviours within romantic relationships. The CBS-R involves assessment of 24 kinds of behaviours in five categories of controlling activities (i.e. economic abuse, coercion and threats, intimidation, emotional abuse, isolation), excluding physical aggression. Women evaluated their own and their partner’s particular kinds of controlling behaviours on a five-point scale (from 0 = never to 4 = always). Only aggregated (overall) CBS-R score was included in the current analysis. Table 1 presents descriptive statistics for CBS-R scores. Cronbach’s alpha for the CBS-R scores was 0.87 for females (self-report) and 0.89 for males (assessment of partner’s controlling behaviour).

**Table 1:** Descriptive statistics for CBS-R scores and MV scores

	Mean	Standard deviation	Median	Minimum-maximum
Overall CBS-R scores				
For females	15.9	10.25	14.0	0–44
For males	13.5	9.44	11.0	1–48
MV score				
For females	23.2	2.63	23.0	13–29
For males	24.0	3.19	24.0	13–29

Mate value (MV) was assessed using a Polish translation of questionnaire proposed by Graham-Kevan and Archer (2009) that has been already used in previous studies (Nowak and Danel 2014; Gomula et al. 2014). Women, compared to other people that they know, rated on a five-point scale (1—very low, 5—very high) both themselves and their partners in terms of six major MV attributes: attractiveness, personality, education, intelligence, career prospects and social status. The significance of particular MV attributes of a potential partner may be different for males and females. For instance, it is well known that a partner’s physical attractiveness is usually more important for men than for women and the cues of resources are more valued by women (Buss 1989). However, there is a consensual agreement between sexes about such desired characteristics in a mate as intelligence, physical attractiveness, health, education and good earning capacity (Buss and Barnes 1986). Furthermore, over the recent decades, male and female preferences became more convergent (Buss et al. 2001). Therefore, the six-item questionnaire used in the study is an adequate and brief measure of the most important aspects of both mate qualities. The scores from the women’s assessments of their own and their partners six characteristics of mate were then averaged, which yielded two aggregated indices of MV separately for women and their partners. Descriptive statistics are presented in Table 1. Cronbach’s alpha for the MV scores was 0.70 for females (self-report) and 0.74 for males (assessment of partner’s MV).

The MVD was defined by subtracting the average (for all six items) woman’s MV from the average MV of her partner. We defined three categories of relationships (following Nowak and Danel 2014): (i) ‘man better’ where a woman assessed her MV lower than her partner’s MV (*n* = 77, 58.33 %), (ii) ‘partners equal’ with no difference between partners’ MVs (both partners MVs were exactly equal, *n* = 22, 16.67 %) and (iii) ‘woman better’ where a woman’s MV was higher than her partner’s MV (*n* = 33, 25.0 %).

### Statistical analysis

We employed mixed-design analysis of variance (split-plot ANOVA), with female CBS self-assessment and her evaluation of the partner’s controlling behaviour intensity as a within-subject repeated measure factor (CONTROL), three MVD categories of relationships (i.e. woman better, partners equal, man better) as between-subject variable and relationship length as a covariate. In order to meet the assumptions of the statistical model (i.e. homogeneity of variances and normal distribution of data), both self-assessed CBS, partner’s CBS and relationship length were log-transformed. Since the CBS scores may have included 0 (response scale was from 0 to 4) and, in such cases, the logarithm is undefined, each CBS score was increased by 10 before transformation (please note that throughout the “Results” section, transformed values are always reported). Further examination of the mixed-design model assumptions confirmed homogeneity of regression slopes and variance-covariance homogeneity. Post hoc tests were used to reveal possible differences in overall controlling behaviour intensity between compared groups of romantic relationships. Additionally, to test directly our predictions, a planned contrast analysis was performed.

The proposed classification of the relationships according to the simple arithmetic differences (or their lack) between both partners’ MVs was convenient and easy for interpretation method of dividing the whole data set into three distinct categories. However, the effect of MVD can be also analysed as a continuous variable. Therefore, in order to validate our results from split-plot ANOVA, we replicated analysis using multivariate multiple regression model with both CBS scores (for woman and her partner) as dependent variables and MVD (continuous variable) and relationship length as predictors. General patterns of result obtained from two analytical approaches were analogous. Thus, later in the article, we report only the outcomes from the analysis based on relationships classified according to MVD categories.

A *p* value of <0.05 indicated statistically significant results. All statistical analyses were conducted in STATISTICA, version 10 (www.statsoft.com).

## Results

Statistical analysis revealed that overall intensity of controlling behaviour (combined effect of the CBS self-assessment and female assessment of her partner CBS, i.e. CONTROL) was significantly different in three groups of relationships (*F*(2,129) = 3.78, *p* = .03, ηp2 = .06). The CONTROL was the highest in woman-better relationships (*M* = 3.27, *SE* = .06), moderate in partners-equal couples (*M* = 3.16, *SE* = .07) and the lowest in man-better pairs (*M* = 3.07, *SE* = .04). Post hoc comparisons with the HSD Tukey’s test showed that the difference between woman-better and man-better relationships is statistically significant (*p* = .03). Other differences did not reach the significance level (i.e. woman-better vs. partners-equal, *p* = .64; man-better vs. partners-equal, *p =* .49).

The covariate, relationship length, was significantly related to the intensity of controlling behaviour (*F*(1,129) = 7.56, *p* = .007, ηp2 = .06). Closer examination revealed that an increase in relationship length was associated with both an increase in the women’s self-assessed CBS (*β* = 0.24, *t*(129) *=* 2.89, *p* = .005) and an increase in the women’s assessment of the partner’s CBS (*β* = 0.20, *t*(129) *=* 2.32, *p* = .02).

**Fig. 1:**
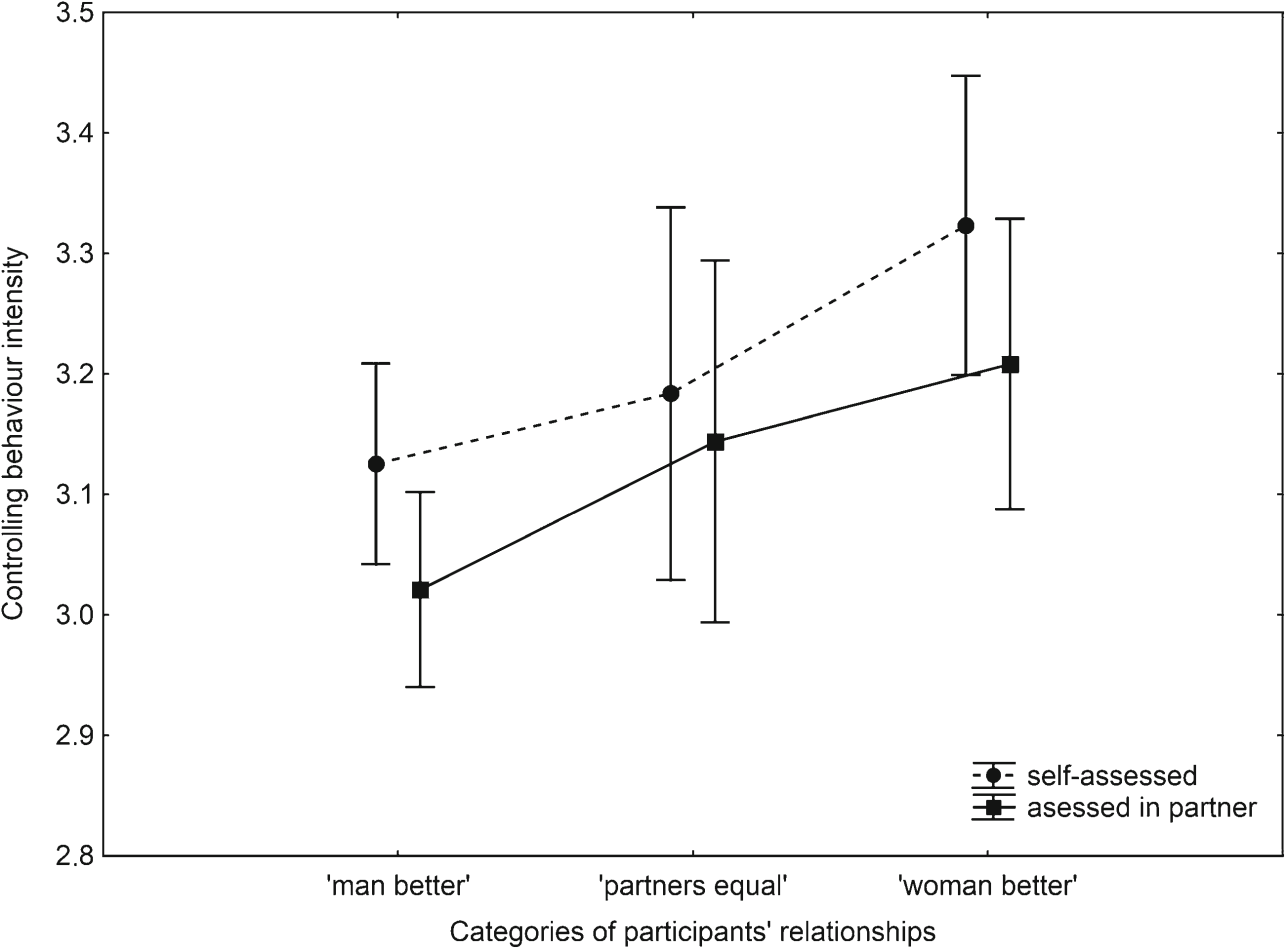
General pattern of the results. *Dots* and *squares* indicate expected means of controlling behaviour intensity calculated for the covariate (relationship length) mean of *M* = 3.37. Please note that all values are transformed (details in the main text). *Whiskers* indicate ±standard error (SE)

The general intensity (regardless of the relationship type) of controlling behaviour did not differ significantly whether self-assessed or estimated in a participant’s partner (*F*(1,129) = .08, *p* = .77). Similarly, non-significant were the effects of interactions between controlling behaviour assessment type (self vs. partner) and relationship length (*F*(1,129) = 1.02, *p* = .31) as well as three categories of women’s relationship (*F*(2,129) = .81, *p* = .44). These results indicated that neither relationship length nor categories of relationship affected the way how a woman assessed her own and her partner’s controlling behaviour. Nonetheless, in order to test our specific predictions regarding controlling behaviours in MV discrepant pairs, the interaction between CONTROL and relationship type (Fig. 1) was further analysed by planned contrast. Contrary to our predictions, the women mated with lower-MV partner used more controlling behaviours (*M* = 3.32, *SE* = .06) than women in men-better (*M* = 3.13, *SE* = .04) relationships (*F*(1,129) = 6.83, *p* = .01). However, as expected, women assessed that men in woman-better relationships (*M* = 3.21, *SE* = .06) used more partner control than men in men-better couples (*M* = 3.02, *SE* = .04; *F*(1,129) = 6.49, *p* = .01). Our prediction that in MVD pairs, it is the lower-MV partner who performs controlling behaviour with greater intensity was supported only for men-better relationships. In such couples, women controlled their MV-better partners more intensively (women: *M* = 3.13, *SE* = .04 vs. men: *M* = 3.02, *SE* = .04; *F*(1,129) = 15.46, *p* < 0.001). Contrary to expectation, women also reported using more partner control than their partners if their MV was higher than their partners’ MV, i.e. in woman-better relationships (women: *M* = 3.32, *SE* = .06 vs. men *M* = 3.21, *SE* = .06; *F*(1,129) = 8.44, *p* = 0.004). In MV-equal pairs, intensities of partner’s control in both sexes were similar (women: *M* = 3.18, *SE* = .08 vs. men: *M* = 3.14, *SE* = .08; *F*(1,129) = .65, *p* = .42).

## Discussion

In this study, we analysed women’s perception of the intensity of controlling behaviours demonstrated by themselves and their partners. We have focused on three types of romantic relationships defined according to the women’s assessment of the difference between their own and their partner’s MV. Our results show that the intensity of controlling behaviours performed by both partners is the highest in relationships perceived by women as a woman-better type (i.e. in those where a woman’s self-reported MV is higher than MV assessed for her partner) and significantly different than in man-better relationships where the intensity of partner control is the lowest. Moreover, on a general level (i.e. regardless of the relationship category), our results show that the intensities of partner control exhibited by women and their partners are similar. These findings are consistent with the results obtained by Graham-Kevan and Archer (2009) and add to the evidence that partner control in humans may be equally important for both sexes. However, a closer examination showed that women’s assessments of their own use of controlling behaviour in MVD couples differed significantly in terms of frequencies of behaviours from the corresponding assessments of their partners’ controlling behaviours; i.e. women report using more controlling behaviour than their partners. Interestingly, in MV-equal pairs, the both frequencies of controlling behaviours were similar. Whereas the greater women’s control of high-MV men supports the idea that controlling behaviour may have evolved as a protection against partner loss, more intense control of lower-quality mate by high-MV women is intriguing. We discuss the obtained results from two perspectives. First, we analyse women’s perception of men’s control of high-quality female partners. Second, we discuss higher intensity of women’s partner control over low MV males. Additionally, we interpret the role or relationship length in controlling behaviours.

### Men’s control of high-quality female partners

As predicted, the perceived level of controlling behaviour performed by male partners is the highest in relationships where women have a higher MV than men. This would suggest that a higher intensity of mate guarding in the lower-MV male partner could be performed in order to avoid high-quality-partner loss and/or mislead parental investments in a sexual rival’s offspring. These results are in accordance with other studies where it has been shown that men involved in a relationship with women having a higher MV than themselves (i.e. relatively younger and more attractive) were more prone to undertake greater mate retention efforts (Buss and Shackelford 1997). Although specific tactics used by lower-MV men did not include direct guarding (putatively the most similar to controlling behaviours), they did involve other negative forms of mate retention, such as emotional manipulation and commitment manipulations (Buss and Shackelford 1997; Shackelford et al. 2005). Similarly, other studies also showed that the negative mate retention tactics, such as direct guarding, partner-directed verbal insults or controlling behaviours per se, are less often performed by higher-quality males (Miner et al. 2009a, b; Graham-Kevan and Archer 2009). Results from our study corroborate the previously mentioned findings and indicate that in relationships where a woman’s MV exceeds a man’s MV, a higher intensity of negative forms of male partner mate guarding could be expected.

### Women’s control of low-quality male partners

The results of this study show that women in woman-better relationships are more prone to control their partners than women in men-better relationships, which is opposed to our predictions and seems to be intriguing from the evolutionary standpoint. Indeed, maintaining and possibly securing the relationship with a lower-MV male partner via controlling tactics are expected to be less beneficial to women in terms of maximizing their fitness. A possible explanation for this unexpected result may stem from the specific nature of woman-better relationships. According to this and previous studies conducted on separate groups of subjects (Nowak and Danel 2014), the majority of women evaluate their own MV as lower when compared to their partner’s MV. This may reflect well-documented women’s preferences for characteristic markers of male MV, such as high social, financial status or career prospects (Buss 1989; Buss and Barnes 1986). Items describing these traditionally ‘male’ qualities of MV constituted a substantial part of the questionnaire used in this study, and although preferences of both sexes have become more convergent (Buss et al. 2001), the overall MV assessment might have been male biased. Moreover, Gomula et al. (2014) have shown that men who perceive their MV as lower than their partner MV (woman-better case) assess their sociosexuality lower (i.e. more restricted and focused on emotional, long-term bonding). This indicates that these men when compared to others have a more feminine, restricted type of sociosexuality. Accordingly, we suggest in our study that relationships where women assess their own MV as higher than their partner’s MV may be those in which traditional gender roles are expressed less explicitly. If so, such females can demonstrate a more masculine pattern of behaviours, and therefore, they control partners more intensively. This is concordant with a general observation suggesting that, in particular societies, the level of aggression between partners is related to the social roles of males and females (Archer 2009). In an individualistic population where gender roles are more equal, victimization of both sexes is more similar as compared to the collectivistic cultures where it is more male biased (Archer 2006). We believe that contemporary Polish population represents rather individualistic than collectivistic model of the society where evolutionary-shaped gender roles may have become overshadowed by cultural influences. As such, the results of our study suggest that the fading importance of traditional gender roles may result in higher women’s partner control, which is in line with other studies conducted on young, well-educated females (Blickenstaff 2005).

An alternative explanation of the higher level of women’s self-reported intensity of controlling behaviour in woman-better relationships may be related to women’s reactions to a partner’s behaviour. Such effect may be a derivative of positive assortative mating (pairing with relatively similar partners) in terms of various psychological characteristics in humans (e.g. Le Bon et al. 2013; Figueredo et al. 2006; Escorial and Martin-Buro 2012; Parshikova et al. 2014). It is also feasible to assume that controlling behaviours used by women in woman-better couples do not serve purely as a mate retention strategy. For example, since high-MV women may have higher standards in their partners (Regan 1998) and CBS questionnaires used in the current study cover also abusive attitudes and behaviours (Graham-Kevan and Archer 2005), high-MV women may use such strategies in order to terminate a disadvantageous relationship with a lower-MV partner. Nonetheless, it is also possible that higher intensity of partner control reported by women is merely spuriously reflected in questionnaire responses and does not reflect reality. In particular, in relationships with high intensity of man’s controlling behaviour, women may self-deceive themselves and report the apparent use of such techniques. However, all the previously mentioned hypotheses (i.e. ‘positive assortative mating for controlling behaviour’, ‘partner drive-away strategy’ and ‘self-deception effect’) should be tested explicitly in further research.

### Relationship length and controlling behaviours

Our results also point out one possible factor that may modify the relationship between partners’ MVD and the intensity of their controlling behaviours. Consistent with our predictions, increased length of relationship was associated with higher intensity of controlling behaviour performed by both partners. Potential loss of relationship and parental investments resulting from a partner’s infidelity or relationship dissolution could be substantially more detrimental in long-term relationships than in newly formed ones. Although a long-term relationship breakdown may be particularly costly for females whose parental efforts are much higher and their reproductive potential is more limited compared to men (review and discussion, e.g. Andersson 1994; Barrett et al. 2002; Cartwright 2000; Danel and Pawłowski 2009; Krebs and Davies 2001; Trivers 1972), males also risk a substantial loss of invested resources in the case of being abandoned by a long-term partner. In such perspective, the higher intensity of mutual use of the controlling tactics, relatively less costly for the performer and therefore possible to use over longer period of time, may be a strategy that counteracts squandering common investments in a long-lasting relationships.

### Conclusions

In conclusion, results from the present study show that the intensity of controlling behaviours performed by both partners is the highest in couples where a woman assesses her own MV as higher than her partner’s MV. In relationships with such MV discrepancies, women experience more often control from their partners and, at the same time, report to control their partners more intensively. Whereas the former result is congruent with evolutionary logic regarding the interaction between partners’ MV and controlling behaviour, the latter is intriguing from the evolutionary perspective of formation and maintenance of human relationship. Overall, our study also implies that the MVD may affect sex differences in the intensities of the mate retention strategies and provides evidence supporting the significance of the relationship length for controlling behaviour intensity.
